# Role of transforming growth factor-β_1_ in down-regulating TNF production by alveolar macrophages during asbestos-induced pulmonary fibrosis

**DOI:** 10.1155/S0962935196000063

**Published:** 1996-02

**Authors:** Irma Lemaire, Sophie Ouellet

**Affiliations:** 1 Laboratory of Immunopharmacology Department of Pharmacology Faculty of Medicine University of Ottawa Ottawa K1H 8M5 Canada

## Abstract

Activation of alveolar macrophages (AM) for tumour necrosis factor production is suppressed initially during the inflammatory response to fibrogenic dusts. We investigated the mechanisms involved in TNF suppression, notably the role of other AM-derived mediators including prostaglandin E_2_ (PGE_2_), transforming growth factor-β_1_ (TGF-β_1_), and interleukin 6 (IL-6). The action of PGE_2_ and TGF-β_1_, on AM was different. At physiologically relevant doses (25–300 pg/ml), PGE_2_ did not cause significant inhibition of Hpopolysaccharide (Lps)-induced TNF release by AM *in vitro* but stimulated IL-6 (up to six fold), an inhibitor of AM-derived TNT. In contrast, TGF-β_1_ (0.5–50 ng/ml) inhibited both LPS-induced TNT and IL-6 release by 50% but had no effect on PGE_2_ production by AM. To determine the respective contribution of these different inhibitors in TNF suppression, AM from rats exposed to fibrogenic asbestos for weeks were treated with neutralizing antibody against TGF-β_1_ or indomethacin, an inhibitor of PGE_2_ synthesis. Treatment of rat AM with anti-TGF-β_1_ but not indomethacin, abrogated the observed TNT suppression. These results suggest that an autocrine, TGF-β_1_-dependent mechanism is involved in the down-regulation of TNF production by rat AM from animals with lung fibrosis.


Mediators of Inflammation 5, 37–42 (1996)

### Role of transforming growth factor-$\beta_1$ in down-regulating TNF production by alveolar macrophages during asbestos-induced pulmonary fibrosis

Irma Lemaire[CA] and Sophie Ouellet

Laboratory of Immunopharmacology, Department of Pharmacology, Faculty of Medicine, University of Ottawa, Ottawa, Canada K1H 8M5.
Fax: (+1) 613 5625456.

[CA]Corresponding Author

**Activation of alveolar macrophages (AM) for tumour necrosis factor (TNF) production is suppressed initially during the inflammatory response to fibrogenic dusts. We investigated the mechanisms involved in TNF suppression, notably the role of other AM-derived mediators including prostaglandin $E_2$ ($PGE_2$), transforming growth factor-$\beta_1$ (TGF-$\beta_1$), and interleukin 6 (IL-6). The action of $PGE_2$ and TGF-$\beta_1$, on AM was different. At physiologically relevant doses (25–300 pg/ml), $PGE_2$ did not cause significant inhibition of lipopolysaccharide (LPS)-induced TNF release by AM *in vitro* but stimulated IL-6 (up to six fold), an inhibitor of AM-derived TNF. In contrast, TGF-$\beta_1$ (0.5–50 ng/ml) inhibited both LPS-induced TNF and IL-6 release by 50% but had no effect on $PGE_2$ production by AM. To determine the respective contribution of these different inhibitors in TNF suppression, AM from rats exposed to fibrogenic asbestos for 3 weeks were treated with neutralizing antibody against TGF-$\beta_1$ or indomethacin, an inhibitor of $PGE_2$ synthesis. Treatment of rat AM with anti-TGF-$\beta_1$ but not indomethacin, abrogated the observed TNF suppression. These results suggest that an autocrine, TGF-$\beta_1$-dependent mechanism is involved in the down-regulation of TNF production by rat AM from animals with lung fibrosis.**

**Key words:** Alveolar macrophages, Fibrosis, Lung inflammation, Prostaglandin $E_2$, Transforming growth factor-$\beta$

### Introduction

Alveolar macrophages (AM) are an important source of numerous cytokines[1] and are a primary cell type in chronic inflammatory reactions induced by fibrogenic dusts.[2] It is likely that the contribution of AM-derived cytokines plays an important role in the maintenance and progression of pulmonary inflammation. Among these, tumour necrosis factor-$\alpha$ (TNF) has attracted much attention owing to a wide range of activities[2] including fibroblast growth-promoting and growth-inhibiting activities.[4] Together with other cytokines, TNF is frequently found at sites of inflammatory reactions and higher levels of TNF messenger RNA were identified in lung tissue of mice treated with silica[5] and bleomycin.[6] With regard to basal and LPS-stimulated TNF production by AM, variable responses including stimulation as well as inhibition or no change have been reported in experimental silicosis.[7–10] Less information is available concerning the contribution of AM-derived TNF in asbestos-induced fibrosis. In a previous study, we reported bidirectional changes in LPS-stimulated TNF production by rat AM exposed to fibrogenic asbestos.[9] These changes were characterized initially by a 50% suppression of TNF and were associated with the development of lung fibrosis but were absent in resolving lung granuloma.[11] TNF inhibition was observed at a time when the bronchoalveolar cell population was composed exclusively of macrophages, raising the possibility of a macrophage-related inhibitory mechanism(s). Therefore, the present work was undertaken to study the role of macrophage-derived mediators, notably $PGE_2$ and TGF-$\beta_1$, in the observed TNF suppression. We report here that down-regulation of AM-derived TNF production was completely abrogated by TGF-$\beta_1$ neutralization whereas indomethacin treatment had no effect. These results suggest that TNF response in AM from animals with lung fibrosis is inhibited in an autocrine fashion by TGF-$\beta_1$.

### Materials and Methods

*Induction of lung inflammation and fibrosis:* Male Wistar rats weighing 225–250 g were pur-

© 1996 Rapid Science Publishers chased from Charles River Canada, Inc. (St Constant, Québec). These animals were derived from a pathogen-free colony, shipped behind filter barriers, and housed in isolated temperature-controlled quarters in an animal isolator unit (Johns Scientific Inc., Toronto). Lung inflammation and fibrosis were induced by intratracheal instillation of a preparation of chrysotile asbestos fibres as described previously.[9] UICC Canadian chrysotile B asbestos fibres, (21% > 10 μm)[12] were obtained from the National Research Institute for Occupational Diseases, Johannesburg, South Africa. Asbestos fibres were autoclaved for 45 min and suspended in sterile phosphate-buffered saline (PBS, pH 7.4) with a Dounce glass homogenizer (Fisher Scientific, Ottawa) before instillation into the animals. Under anaesthesia, the trachea was exposed surgically and saline or chrysotile B fibres were briskly injected through an 18-gauge needle. Two groups of seven rats each, received respectively, a single intratracheal injection of saline (control) or chrysotile B (5 mg). The rats from each group were sacrificed 3 weeks after treatment and were analysed by bronchoalveolar lavage (BAL), for BAL cell populations and AM-derived TNF production. Histological examination of lung sections stained with haematoxylin–eosin or Masson's trichrome revealed the presence of fibrosis in the lungs of rats exposed to UICC chrysotile fibres.[9] The lesions consisted of granulation tissue with fibroblastic proliferation and collagen deposition and were localized predominantly in and around terminal bronchioles.

*Alveolar macrophage culture:* Alveolar macrophages were obtained by bronchoalveolar lavage as described previously.[9] Cells were counted in a haemocytometer chamber and viability (98–100%) was determined by trypan blue exclusion. Differential analysis of lavage cells made from cytocentrifuge smears (Shandon, $2.5 \times 10^4$ cells) and stained with Wright–Giemsa indicated that the BAL cell population was essentially composed of macrophages (99% AM) in normal rats. Similarly, in saline and asbestos-treated rats, the BAL cell population was 99% AM, the major difference between these two groups being a significant increase in AM number in asbestos-treated animals ($11 \times 10^6$ and $19 \times 10^6$ respectively, for saline and asbestos groups). For determination of TNF, IL-6 and TGF-$\beta_1$ bioactivities, AM ($0.2 \times 10^6$) from normal rats and when indicated, from saline and asbestos-treated rats, were incubated in 0.2 ml of DMEM supplemented with 0.5% FBS for 18 h at 37°C in the presence and absence of LPS (1 μg/ml) (Sigma Chemical Co., St Louis, MO). Culture supernatants were collected by centrifugation and frozen at −80°C until assayed.

*Assays of TNF, IL-6 and TGF-$\beta_1$:* TNF activity was determined as described[9] using the L929 murine fibroblast lysis assay. Serial dilutions of test supernatant were added to $4.5 \times 10^4$ L929 cells in the presence of actinomycin D (1 μg/ml) (Boehringer Mannheim) and incubated for 18 h in microtitre plates. The supernatants were discarded; the remaining adherent viable cells were stained with crystal violet (0.5% in 2% methanol) and the absorbance of each well was read at 540 nm using an automated Bio-Tek microplate reader (Mandel Scientific). Each assay was standardized with murine recombinant TNF ($4 \times 10^7$ U/mg, Genzyme) and TNF units were calculated by probit analysis. TNF bioassay was validated by using a rabbit antimurine TNF antibody (Genzyme Corporation, Boston, MA) which completely neutralizes the cytotoxicity of AM conditioned media.

IL-6 activity was measured with the standard B9 cells proliferation assay as described.[13] B9 cells ($0.5 \times 10^4$) were incubated in 0.2 ml of Iscove's Modified Dulbecco's Medium (IMDM) (Grand Island Biological Co.) supplemented with 5% FBS and $5 \times 10^{-5}$ M 2-mercaptoethanol in the presence of AM supernatants at various dilutions. Tritiated thymidine (1 μCi/well) was added after 66 h of incubation and cultures were harvested at 72 h with a Skatron filtration device. The sample dilution curve was related to a standard curve generated with recombinant murine IL-6 (Genzyme, $10^8$ U/mg) and IL-6 units were calculated by probit analysis.

TGF-$\beta_1$ activity was determined using the selectively sensitive Mv1Lu, mink lung connective tissue cell line (American Type Culture Collection, Rockville, MD; no. CCL-64) as described.[14] Cells ($2.5 \times 10^4$/well) were cultured for 4 h in 96-well flat-bottomed microtitre plates (Costar, Cambridge, MA) in MEM supplemented with 10% foetal bovine serum (FBS) (Grand Island Biological Co., Grand Island, NY). AM culture supernatants were then added in triplicate and incubation was performed for 18 h. Cells were pulsed for 6 h with [$^3$H]thymidine (1.5 μCi/well) (Dupont NEM). After washing with PBS, cells were treated with trypsin–EDTA (0.5–0.03%) for 40 min at room temperature and collected with a Skatron harvester. Results were calculated based on the decrease of [$^3$H]thymidine incorporation compared with natural human TGF-$\beta_1$ standard (Upstate Biotechnology Inc., NY) and data are expressed as ng/ml. The specificity of the assay was demonstrated by using chicken anti-human TGF-$\beta_1$ (R & D Systems) which inhibits the activity.

*Prostaglandin $E_2$ determination:* Prostaglandin $E_2$ ($PGE_2$) was determined from AM supernatants using an ELISA kit (Cayman Chemical, Ann Arbor, MI). The ELISA was a competitive acetylcholinesterase-linked immunoassay and was performed according to the manufacturer's instruction.

*Neutralization and blocking experiments:* AM obtained from animals exposed to saline or UICC chrysotile B asbestos for 3 weeks were incubated as follows: AM ($10^6$/ml) from each group were incubated for 18 h at 37°C with LPS (1 µg/ml) alone or in the presence of a purified turkey IgG antibody (10 µg) to human transforming growth factor-$\beta_1$ (anti-TGF-$\beta_1$) which displays neutralizing activity against rat TGF-$\beta_1$ (Collaborative Research, Bedford MA), turkey serum as a control for anti-TGF-$\beta_1$ or indomethacin ($10^{-5}$ M) (Sigma, St Louis, MO). AM culture supernatants were collected and frozen at −80°C for TNF measurement as described above.

*Statistical analysis:* Results were expressed as mean values ± S.E.M. Statistical significance of differences between treated and control groups were determined using a one-way analysis of variance and Bonferrani test (Instat) ($p < 0.05$).

### Results

$PGE_2$ inhibited LPS-induced TNF release from AM by 50% in a dose-dependent manner with half-maximal effect observed at 25 µg/ml (Fig. 1A). This is much higher than the levels of $PGE_2$ produced by LPS activated AM (805 pg/ml) (Table 1) indicating that physiological concentrations of $PGE_2$ are not sufficient to inhibit TNF production by rat AM. In contrast, relatively small concentrations of $PGE_2$ (25–300 pg/ml) had a direct stimulatory effect on spontaneous IL-6 release (7 U/ml), an inhibitor of TNF production by rat AM.[11] Up to six-fold stimulation (46 U/ml) was obtained with 300 pg/ml $PGE_2$ (Fig. 1B). In addition, LPS-stimulated IL-6 release (75 U/ml) was further increased two- to five-fold by $PGE_2$ within the same concentration range.

On the other hand, TGF-$\beta_1$ at concentrations ranging between 0.5 and 50 ng/ml caused significant inhibition of LPS-induced TNF (Fig. 2A) and IL-6 (Fig. 2B) release by rat AM with maximal inhibition (50%) obtained with 2.5–5 ng/ml. In addition, TGF-$\beta_1$ had a potent inhibitory effect (54%) on LPS-induced $PGE_2$ production by AM (Table 1). Thus, TGF-$\beta_1$ can directly inhibit TNF production by AM whereas $PGE_2$ may indirectly down-regulate TNF by stimulating IL-6.

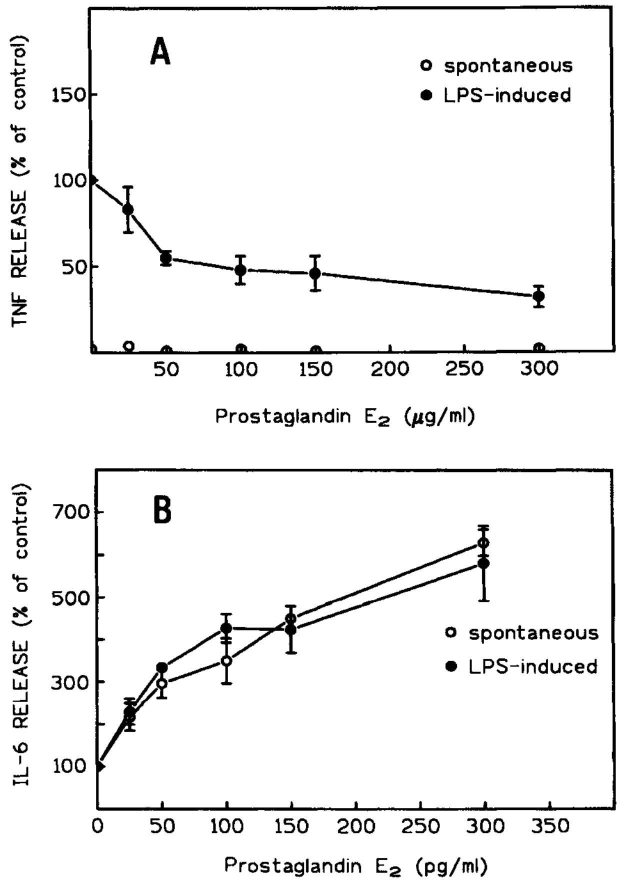

FIG. 1. AM ($10^6$/ml) from normal rat were incubated with various concentrations of $PGE_2$ as indicated, for 18 h in the presence and absence of LPS (1 µg/ml). (A) TNF activity was measured in AM culture supernatants as described in Materials and Methods. AM + LPS = 134 U/ml or 3350 pg/ml. Values represent mean ± S.E.M. of three separate experiments. (B) IL-6 activity was determined in AM culture supernatants as described in Materials and Methods. Spontaneous IL-6 release (100% response) was equivalent to 7 U/ml and $PGE_2$ stimulatory effect was expressed as percent of control. LPS-induced IL-6 release (100% response) was equivalent to 75 U/ml or 750 pg/ml and $PGE_2$ stimulatory effect was expressed as percent of control. Values represent mean ± S.E.M. of four separate experiments.

**Table 1.** Effects of TGF-$\beta_1$ on $PGE_2$ production by AM

| Treatment | $PGE_2$ (pg/ml)[a] |
|---|---|
| AM | 158.5 ± 54 |
| AM + LPS (1 µg/ml) | 805.2 ± 72 |
| AM + TGF-$\beta_1$ (2 ng/ml) | 179.8 ± 37 |
| AM + LPS + TGF-$\beta_1$ (2 ng/ml) | 378 ± 31 |

[a]Alveolar macrophages ($10^6$/ml) were incubated as described in Materials and Methods for 18 h in media alone or in the presence of LPS or TGF-$\beta_1$ or a combination of both. Prostaglandin concentrations were determined in culture supernatants by ELISA. Data represent mean ± S.E.M. of three separate experiments.

To investigate which pathway(s) may be involved in the down-regulation of AM-derived TNF following asbestos exposure, AM obtained from rats exposed to UICC chrysotile asbestos

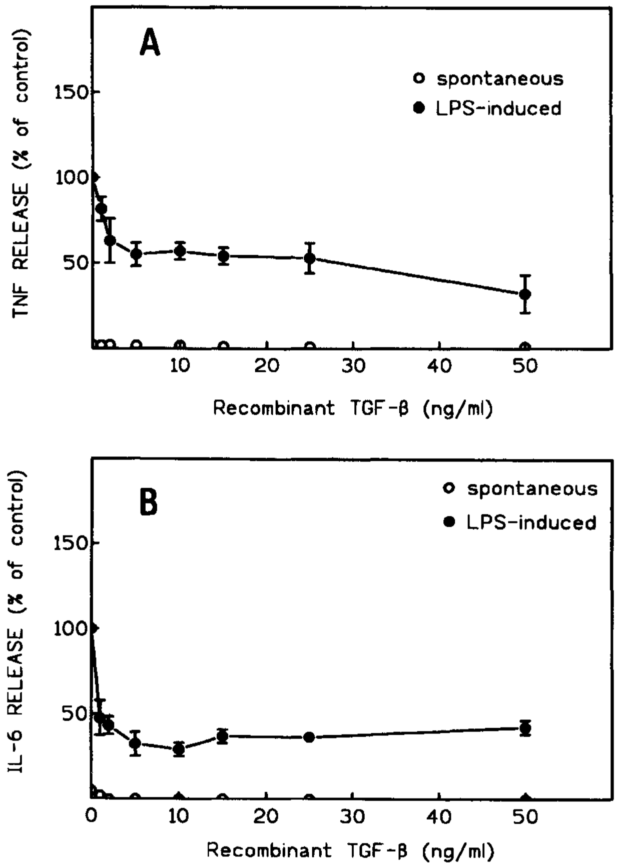

FIG. 2. AM ($10^6$/ml) were incubated with increasing concentrations of recombinant TGF-$\beta_1$ for 18h in the presence and absence of LPS (1 μg/ml). (A) TNF activity was measured in AM culture media as described in Materials and Methods. AM+LPS=125U/ml or 3125pg/ml. Values represent mean ± S.E.M. of three separate experiments. (B) IL-6 activity was measured in AM culture media as described in Materials and Methods. AM+LPS=72U/ml or 720pg/ml. Values represent mean ± S.E.M. of four separate experiments.

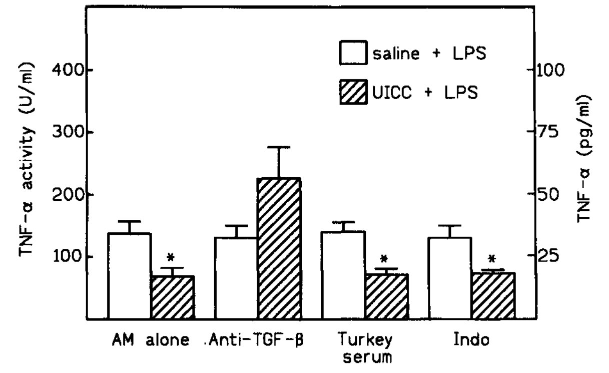

FIG. 3. AM ($10^6$/ml) from rats treated with saline or UICC chrysotile B asbestos (5mg) for 3 weeks were incubated for 18h in the presence of LPS (1 μg/ml) with either anti-TGF-$\beta_1$, turkey serum or indomethacin. TNF activity was determined in AM culture supernatants as described in Materials and Methods. Values represent mean ± S.E.M. of five animals per group. *Significantly different from control at $p < 0.05$.

**Table 2.** Production of TGF-$\beta_1$ by AM from control and asbestos treated rats

| Treatment | TGF-$\beta_1$ (ng/ml)[a] |
|---|---|
| Saline | 0.16 ± 0.03 |
| Asbestos | 1.2 ± 0.1 |

[a]Alveolar macrophages ($10^6$/ml) from rats treated with saline or asbestos for 3 weeks, were incubated for 18h. Culture supernatants were collected and measured for TGF-$\beta_1$ as described in Materials and Methods. Values represent mean ± S.E.M. of five animals per group.

for 3 weeks were incubated in the presence and absence of anti-TGF-$\beta_1$ known to neutralize rat TGF-$\beta_1$,[14] turkey serum as a control, and indomethacin, an inhibitor of $PGE_2$ synthesis. Treatment with anti-TGF-$\beta_1$ but not with turkey serum or indomethacin abrogated the 50% suppression of AM-derived TNF seen 3 weeks after asbestos exposure (Fig. 3). At this time, AM culture media from asbestos exposed rats contained levels of TGF-$\beta_1$ (1.2ng/ml) sufficient for inhibition of TNF release (Table 2).

### Discussion

We have investigated the mechanisms involved in TNF suppression during the development of asbestos-induced fibrosis,[9] at the macrophage level. A variety of mediators are known to inhibit TNF production from cells including IL-4,[15] IL-6,[16] IL-10,[17] $PGE_2$[18] and TGF-$\beta_1$.[19] Among these, IL-4 is produced by activated T-lymphocytes,[20] a cell type which is not present in the bronchoalveolar compartment of our inflammatory lung model.[2,11] Because the alveolar macrophage (AM) is a prominent feature of lung reactions to asbestos,[2] our study focused on mediators produced by AM, notably $PGE_2$, IL-6 and TGF-$\beta_1$. As reported in other systems,[19] TGF-$\beta_1$ exerts significant inhibitory effects on TNF release by activated rat AM, conditions that are consistent with inflammatory states. TGF-$\beta_1$ inhibits TNF release within the concentration range produced by AM suggesting that it could act in an autocrine fashion to modulate TNF production in the pulmonary microenvironment. In addition, TGF-$\beta_1$ inhibits LPS-induced $PGE_2$ indicating that it suppresses TNF production through a $PGE_2$-independent pathway. This is consistent with previous findings in peripheral blood mononuclear cells[19] and murine macrophage cell lines.[21] Furthermore, TGF-$\beta_1$ also inhibits IL-6 release from activated AM, ruling out the contribution of IL-6[16] in mediating its suppressive effect on TNF. Although TGF-$\beta_1$ has been reported to stimulate IL-6 production in fibroblasts,[22] chondrocytes[23] and peripheral blood lymphocytes,[24] it has been shown to suppress IL-6 in bone marrow cells.[25] Such differences are likely to reflect cell-specific regulation of TNF by TGF-$\beta_1$. The molecular basis of TGF-$\beta_1$ inhibitory action on TNF production by rat AM is unknown. As reported for human peripheral blood mononuclear cells[19] and murine peritoneal macrophages,[26] TGF-$\beta_1$ may block TNF by acting post-transcriptionally in rat AM, possibly through increased mRNA degradation or inhibition of TNF mRNA translation.[26] In this respect, and consistent with our observations, TGF-$\beta_1$ appears to act differently to $PGE_2$ and IL-6, which both have been shown to inhibit TNF transcription.[18,27] Further investigation would be necessary to clarify this.

In contrast, relatively high non-physiological concentrations of $PGE_2$ ($10^{-5}$ M range) are necessary for inhibition of TNF. This is in agreement with previous studies which showed AM to be less susceptible than monocytes to $PGE_2$ inhibition requiring much higher concentrations of $PGE_2$ ($10^{-5}-10^{-4}$ M) for suppression of TNF.[28] Such high inhibitory levels of $PGE_2$ could not be achieved following LPS stimulation, indicating that, under our experimental conditions, activated rat AM do not produce enough $PGE_2$ to interfere with TNF production. $PGE_2$ on the other hand, stimulates IL-6 release in rat AM. This is in contrast to a recent report that $PGE_2$ inhibits LPS-induced release of IL-6 in murine peritoneal macrophages.[29] Such discrepancies are likely due to differences in the experimental conditions used, including cell preparations and concentrations of $PGE_2$. As mentioned earlier, AM are fairly resistant to inhibition by $PGE_2$ concentrations ($10^{-9}$ M) used in the latter study.[29] However, our results are in accordance with other studies which demonstrate that augmentation of $PGE_2$ correlates with an increase in IL-6[30] whereas inhibition of $PGE_2$ blocks IL-6 production.[31,32] Interestingly, stimulation of IL-6 by $PGE_2$ is observed within the physiological range of concentrations produced by AM, raising the possibility that, under certain conditions, $PGE_2$ may contribute indirectly to TNF suppression by increasing IL-6 levels. Our study, however, clearly demonstrates that TNF suppression in AM from fibrogenic rats is mediated through a TGF-$\beta_1$-dependent $PGE_2$-independent mechanism(s). This is based on our observations that neutralizing antibody to TGF-$\beta_1$ blocks the observed 50% inhibition of TNF whereas indomethacin treatment has no effect. Furthermore, in contrast to controls, AM from animals exposed to asbestos produce amounts of TGF-$\beta_1$ sufficient to cause inhibition. The contribution of other inhibitors, notably IL-6 and IL-10, cannot be completely ruled out. However, our observations that $PGE_2$ at physiological concentrations stimulate IL-6, do not suggest a role for IL-6. In support of this, previous work from this laboratory demonstrated a lack of correlation between AM-derived IL-6 and TNF production following asbestos exposure.[11] Similarly, since $PGE_2$ deactivation of macrophages has recently been shown to be mediated by IL-10,[29] our results are not consistent with a role for IL-10. Further experiments would be necessary to address these issues.

Collectively, our findings support a role for AM-derived TGF-$\beta_1$ in down-regulating AM-derived TNF in an autocrine fashion. Our study also provides further evidence for important interactions between TGF-$\beta_1$ and TNF during inflammatory responses to injury. In this regard, it is interesting to note that TGF-$\beta_1$ and TNF antagonize each other's functions. Thus, TGF-$\beta_1$ blocks numerous effects of TNF[33] whereas TNF inhibits the growth-promoting effects of TGF-$\beta_1$[34] and wound healing.[35] This is in agreement with recent work which demonstrated that mRNA for TNF was greatly increased in TGF-$\beta_1$ knock-out animals[36] and that deficient expression of TGF-$\beta_1$ was correlated with dysregulated production of TNF and lethal dysfunction of the inflammatory system. Since TGF-$\beta_1$ exerts numerous biological activities that are directly relevant to tissue repair,[37] TGF-$\beta_1$-induced suppression of TNF may be a requisite step for appropriate repair response following injury.

### References

1. Fels AOS, Cohn ZA. The alveolar macrophage. *J Appl Physiol* 1986; **60:** 353–369.
2. Lemaire I. Silica- and asbestos-induced pulmonary fibrosis. In: Phan SH, Thrall RS, eds. *Pulmonary Fibrosis Lung Biology in Health and Disease.* New York: Marcel Dekker, 1995; 319–362.
3. Old LJ. Tumor necrosis factor. In: Bonavida B, Granger E, eds. *Tumor Necrosis Factor: Structure, Mechanisms of Action, Role in Disease and Therapy.* Basel: Karger, 1990; 1–30.
4. Thorton SC, Por SB, Walsh BJ, Penny R, Breit SN. Interaction of immune and connective tissue cells. I. The effect of lymphokines and monokines on fibroblast growth. *J Leukoc Biol* 1990; **97:** 312–320.
5. Piguet PF, Collart MA, Grau GF, Sappino AP, Vassalli P. Requirement of tumor necrosis factor for development of silica-induced pulmonary fibrosis. *Nature* 1990; **344:** 245–247.
6. Piguet PF, Collart MA, Grau GE, Kapanci Y, Vassalli P. Tumor necrosis factor/cachectin plays a key role in bleomycin-induced pneumopathy and fibrosis. *J Exp Med* 1989; **170:** 655–663.
7. Driscoll KE, Lindenschmidt KC, Maurer JK, Higgins JM, Ridder G. Pulmonary response to silica or titanium dioxide: inflammatory cells, alveolar macrophage-derived cytokines and histopathology. *Am J Respir Cell Mol Biol* 1990; **2:** 381–390.
8. Bissonnette E, Rola-Pleszczinski M. Pulmonary inflammation and fibrosis in a murine model of asbestosis and silicosis. Possible role of tumor necrosis factor. *Inflammation* 1989; **13:** 329–339.
9. Ouellet S, Yang H, Aubin RA, Hawley RG, Wenckebach GFC, Lemaire I. Bidirectional modulation of TNF-α production by alveolar macrophages in asbestos-induced pulmonary fibrosis. *J Leukoc Biol* 1993; **53:** 279–286.
10. Mohr C, Gemsa D, Graebuer C, *et al.* Systemic macrophage stimulation in rats with silicosis: enhanced release of tumor necrosis factor-α from alveolar and peritoneal macrophages. *Am J Respir Cell Mol Biol* 1991; **5:** 395–402.
11. Lemaire I, Ouellet S. Distinctive profile of alveolar macrophage-derived cytokine release induced by fibrogenic and non-fibrogenic mineral dusts. *J Toxicol Environ Health* 1996; **47**: 101–115..
12. Timbrell V. Characteristics of the International Union against Cancer standard reference samples of asbestos. In: *Pneumocomosis*, Proceedings of an International Conference. Johannesburg, 1990; 28–36.
13. Lemaire I, Yang H, Cantin M-F, Lemaire S. Up-regulation of cytokine production in alveolar macrophages by histogranin, a novel endogenous pentadecapeptide. *Immunol Letts* 1994; **41**: 37–42.
14. Lauzon W, Lemaire I. Alveolar macrophage inhibition of lung-associated NK activity: involvement of prostaglandins and transforming growth factor-$\beta_1$. *Exp Lung Res* 1994; **20**: 331–349.
15. Hart PH, Vitti GF, Burgess DR, Whitty GA, Piccoli DS, Hamilton JA. Potential antiinflammatory effects of IL-4: suppression of human monocyte tumor necrosis factor alpha, interleukin-1 and prostaglandin $E_2$. *Proc Natl Acad Sci USA* 1989; **86**: 3803–3807.
16. Aderka D, Lee J, Vilcek J. IL-6 inhibits lipopolysaccharide-induced TNF production in cultured human monocytes, U937 cells and in mice. *J Immunol* 1989; **143**: 3517–3523.
17. Fiorentino DF, Zlotnik A, Mossman TR, Howard M, O'Garra A. Interleukin-10 inhibits cytokine production by activated macrophages. *J Immunol* 1991; **147**: 3815–3822.
18. Kunkel SL, Spengler M, May MA, Spengler R, Larrick J, Remick D. Prostaglandin $E_2$ regulates macrophage-derived tumor necrosis factor gene expression. *J Biol Chem* 1988; **263**: 5380–5384.
19. Chantry D, Turner M, Abney E, Feldmann M. Modulation of cytokine production by transforming growth factor-$\beta_1$. *J Immunol* 1989; **142**: 4295–4300.
20. Howard M, Farrar J, Hilfilker M, *et al.* Identification of a T cell-derived B cell growth factor distinct from interleukin 2. *J Exp Med* 1982; **155**: 914–923.
21. Reddy ST, Gilbert RS, Xie W, Luner S, Herschman HR. TGF-beta$_1$ inhibits both endotoxin-induced prostaglandin synthesis and expression of the TIS10/prostaglandin synthase 2 gene in murine macrophages. *J Leukoc Biol* 1994; **55**: 192–200.
22. Elias J, Leutz V, Cummings PJ. Transforming growth factor-β regulation of IL-6 production by unstimulated and IL-1 stimulated human fibroblasts. *J Immunol* 1991; **146**: 3437–3443.
23. Guerne PA, Carson DA, Lotz M. IL-6 production by human articular chondrocytes. Modulation of its synthesis by cytokines, growth factors and hormones *in vitro*. *J Immunol* 1990; **144**: 499–505.
24. Turner M, Chantry D, Feldman M. Transforming growth factor beta induces the production of interleukin 6 by human peripheral blood mononuclear cells. *Cytokine* 1990; **2**: 211–216.
25. Lotem J, Sachs L. Selective regulation of the activity of different hematopoietic regulatory proteins by transforming growth factor $\beta_1$ in normal and leukemic myeloid cells. *Blood* 1990; **76**: 1315–1322.
26. Bogdan C, Paik J, Vodovotz Y, Nathan C. Contrasting mechanisms for suppression of macrophage cytokine release by transforming growth factor-β and interleukin-10. *J Biol Chem* 1992; **267**: 23301–23308.
27. Schindler R, Mancilla J, Endres S, Ghorbani R, Clark SC, Dinarello CA. Correlations and interactions in the production of interleukin-6 (IL-6), IL-1 and tumor necrosis factor (TNF) in human blood mononuclear cells: IL-6 suppresses IL-1 and TNF. *Blood* 1990; **75**: 40–47.
28. Strieter RM, Remick DG, Lynch JP, *et al.* Differential regulation of tumor necrosis factor-alpha in human alveolar macrophages and peripheral blood monocytes: a cellular and molecular analysis. *Am J Respir Cell Mol Biol* 1989; **1**: 57–63.
29. Strassmann G, Patil-Koota V, Finkelman F, Fong M, Kambayashi T. Evidence for the involvement of interleukin 10 in the differential deactivation of murine peritoneal macrophages by prostaglandin $E_2$. *J Exp Med* 1994; **180**: 2365–2370.
30. Marcinkiewicz J. *In vitro* cytokine release by activated murine peritoneal macrophages: role of prostaglandins in the differential regulation of tumor necrosis factor alpha, interleukin 1 and interleukin-6. *Cytokine* 1991; **6**: 327–332.
31. Stadler J, Harbrecht BG, DiSilvio M, *et al.* Endogenous nitric oxide inhibits the synthesis of cyclooxygenase products and interleukin-6 by rat Kupffer cells. *J Leukoc Biol* 1993; **53**: 165–172.
32. Ertel W, Morrison MH, Wang P, Zheng F, Ayala A, Chaudry IH. The complex pattern of cytokines in sepsis. Association between prostaglandins, cachectin and interleukins. *Annals of Surgery* 1991; **214**: 141–148.
33. Cai JP, Falanga V, Chin YH. Transforming growth factor-β regulates the adhesive interactions between mononuclear cells and microvascular endothelium. *J Invest Dermatol* 1991; **97**: 169–174.
34. Steenfos HH, Hunt TK, Schenenstuhl H, Goodson WH. Selective effects of tumor necrosis factor-alpha on wound healing in rats. *Surgery* 1989; **106**: 171–176.
35. Rapala K, Laato M, Niikoski J, *et al.* Tumor necrosis factor alpha inhibits wound healing in the rat. *Eur Surg Res* 1991; **23**: 261–268.
36. Shull MM, Ormsby I, Kier AB, *et al.* Targeted disruption of the mouse transforming growth factor-β1 gene results in multifocal inflammatory disease. *Nature* 1992; **359**: 693–699.
37. Roberts AB, Sporn MB. The transforming growth factor βs. In: Sporn MB, Roberts AB, eds. *Peptide Growth Factors and their Receptors*. Heidelberg: Springer-Verlag 1990; 419–472.

ACKNOWLEDGEMENTS. This work was supported by grant MT-7310 from the Medical Research Council of Canada (MRC). I.L. is a scholar of MRC (DG-350). The authors thank P. Drouin for competent preparation of figures and C. Lalonde for diligent secretarial help.

**Received 17 October 1995; accepted in revised form 21 November 1995**
